# Health Literacy Co-Design in a Low Resource Setting: Harnessing Local Wisdom to Inform Interventions across Fishing Villages in Egypt to Improve Health and Equity

**DOI:** 10.3390/ijerph18094518

**Published:** 2021-04-24

**Authors:** Wagida A. Anwar, Nayera S. Mostafa, Sally Adel Hakim, Dalia G. Sos, Christina Cheng, Richard H. Osborne

**Affiliations:** 1 Department of Community, Environmental and Occupational Medicine, Faculty of Medicine, Ain Shams University, Cairo 11566, Egypt; wagidaanwar@med.asu.edu.eg (W.A.A.); drsallyhakim7@gmail.com (S.A.H.); daliagabesos@gmail.com (D.G.S.); 2 Centre for Global Health and Equity, Swinburne University of Technology, Melbourne, VIC 3122, Australia; cccheng@swin.edu.au (C.C.); rosborne@swin.edu.au (R.H.O.)

**Keywords:** health literacy, fishermen, co-design, Borollos lake, health literacy questionnaire (HLQ), health inequality, Ophelia (optimising health literacy and access) process

## Abstract

Fishermen in low resource settings have limited access to health services and may have a range of health literacy-related difficulties that may lead to poor health outcomes. To provide solutions and interventions based on their needs, co-design is considered best practice in such settings. This study aimed to implement a co-design process as a step towards developing health literacy interventions to improve health and equity in the Borollos Lake region of northern Egypt, a low resource setting with a high prevalence of chronic diseases. This study was guided by the Ophelia (Optimising Health Literacy and Access) process, a widely used and flexible co-design process that seeks to create local and fit-for-purpose health literacy solutions through genuine engagement and participation of community members and relevant stakeholders. Following a health literacy survey using the Health Literacy Questionnaire (HLQ), cluster analysis was conducted to identify the diverse health literacy profiles among the fishing communities. Seven health literacy profiles were identified. Vignettes, representing these profiles, were presented and discussed in ideas generation/co-design workshops with fishermen and health workers to develop intervention ideas. Seventeen fishermen, 22 wives of fishermen, and 20 nurses participated in four workshops. Fifteen key strategies across five themes, including ‘Enhancing education among fishing communities’, ‘Provide good quality health services’, ‘Financial support for health’, ‘Social support for health’, and ‘Promote better health-related quality of life among fishermen’, were generated. The ideas did not only target the individuals but also required actions from the government, non-government organizations, and fishermen syndicates. By harnessing local wisdom, the Ophelia process has created meaningful engagement with the local communities, leading to a wide range of practical and feasible solutions that match the special needs and environment of a low resource setting.

## 1. Introduction

Fishing is considered one of the most dangerous occupations in the world due to the constant exposure to unpredictable weather, regular use of heavy machinery in unstable environments and long working hours [1,2]. Smoking and poor diet are also among the common behaviour health risks in fishermen [3,4]. The Borollos Lake in northern Egypt, located in Kafr el-Sheikh Governorate east of Rosetta, bordered by the Mediterranean Sea, has many islands separated by great distances inhabited by fishermen communities. These communities are typically poor, with low education (including high rates of illiteracy), and have limited access to health services because of the remote location. Fishermen are at high risk of musculoskeletal disorders, hearing problems, sunburn, physical trauma as well as psychological stress arising from job instability [2,5,6]. Our recent health literacy survey using the Health Literacy Questionnaire (HLQ), a commonly used questionnaire used to support health literacy intervention development, found that people in this region have remarkably low health literacy on most dimensions of health literacy. The findings clearly show that fishermen and their families are experiencing health literacy challenges that will likely lead to poor health outcomes [5] and efforts are needed to improve health and equity outcomes for the fishing communities living in low resource settings.

Literacy varies globally, from a country to another and even in the same country [7]. Health literacy is a multidimensional mechanism that determines people’s knowledge, confidence, and comfort (which accumulate through daily activities, social interactions and across generations) to access, understand, appraise, remember, and use information about health and health care [8]. It is closely linked to health equity and can be used to understand who is missing out on current services, why individuals and groups are being left behind, and how health policy, programs and interventions can be developed and/or improved to accelerate impacts on health and equity [9]. The HLQ, developed using a grounded approach and demonstrated to have robust psychometric properties, measures nine domains of health literacy:

1. Feeling understood and supported by healthcare providers.
2. Having sufficient information to manage my health.
3. Actively managing my health.
4. Social support for health.
5. Appraisal of health information.
6. Ability to actively engage with healthcare providers.
7. Navigating the healthcare system.
8. Ability to find good health information.
9. Understand health information well enough to know what to do [10].

By assessing the nine dimensions of health literacy, the HLQ provides insights into an individual’s experiences when engaging with health information, health practitioners and health services [10], and it provides a comprehensive picture about how individual, social and cultural contexts influence an individual’s health literacy [11]. Through the Ophelia (Optimising Health Literacy and Access) process, the HLQ informs the development and implementation of interventions by healthcare organizations to meet the health literacy needs of their communities.

The Ophelia process is a widely used method developed with community engagement and co-design as the core principles to improve access, equity and outcomes by addressing health literacy needs [12]. The development of interventions to serve community needs must take into consideration the contexts and needs of those living in the community. To achieve this purpose, a co-design approach harnessing local wisdom and collective creativity [13] is considered best practice, especially when working with vulnerable populations [14]. Local wisdom is highly regarded as it refers to the knowledge and values gained through experiences and activities by a group of people. It may pass on from one generation to the next generation. The Ophelia process has been applied mainly in European and Western settings with considerable success, including within the World Health Organization (WHO) National Health Literacy Development Program and in a digital health context [15,16,17,18,19,20,21].

Given the unique features of the fishing communities in the Borollos Lake region of northern Egypt, the importing of externally developed public health interventions, either from the published literature or from other sources, may not match what might be needed and useful for fishermen and their families. Consequently, we applied the Ophelia process to provide in-depth assessment of community needs and deep community engagement in the context of fishing village life for the development of potentially useful interventions.

The aim of this study was to implement the co-design process where university staff engaged with fishermen and healthcare provider representatives through the Ophelia process to develop health literacy interventions that meet the needs of fishing communities, with the ultimate aim to improve health and equity outcomes.

## 2. Materials and Methods

**Figure 1:**
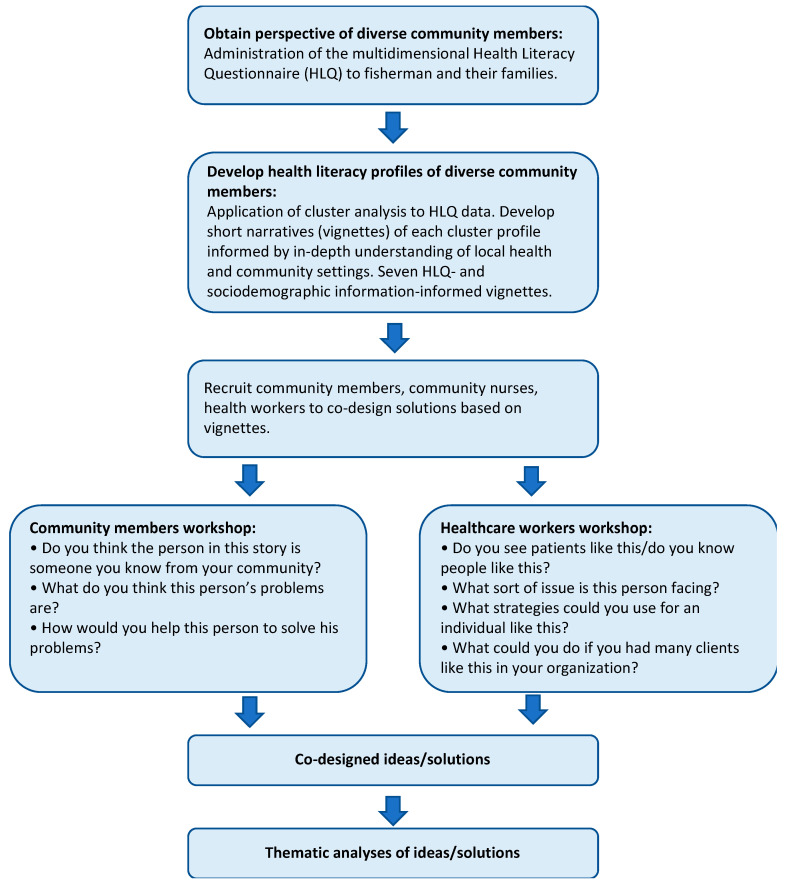
Study process.

This study was set in the remote Borollos Lake region in Kafr El Shiekh, Egypt and the methods were guided by the Ophelia process. This process was developed based on intervention mapping, quality improvement collaboratives and realist synthesis to improve individual and organizational health literacy responsiveness [12]. The process includes three phases: (1) identifying local needs; (2) co-design of interventions; and (3) implementation, evaluation and ongoing improvement [12,21]. This study represents phase 1 of the Ophelia process, which involves three steps: (1) health literacy survey; (2) cluster analysis and develop vignettes; and (3) ideas generation/co-design workshops. See Figure 1 of the study process.

The first step of health literacy survey was conducted from January–May 2018 and the results are described elsewhere [5]. Study data is available in the Supplementary Material. In summary, data were collected from five villages where the majority of their inhabitants were fishermen. The socio-demographic data collected included age, sex, living alone or with others, internet usage, family income, occupation (fisherman or other), educational attainment (illiterate, primary level, or above primary level) [5]. A total of 436 participants, including fishermen and their families, completed the survey by face-to-face interview. The age range was 18–89 with 65.2% aged under 50 years and 50% were males. Over a third of the sample (37.1%) were illiterate and 42.4% were active fishermen. The results showed that they had relatively low to very low scores for most of the health literacy dimensions but had some strengths in terms of social support and communication with health professionals [5]. See Table 1.

### 2.1. Cluster Analysis and Vignette Development

The cross-sectional survey provided a glimpse of the health literacy of fishermen and their families. Given the diverse demographic characteristics which may lead to different health literacy strengths or weaknesses among subgroups within the sample, the Ophelia process recommends using cluster analysis, based on the nine scale scores of the HLQ, to identify groups with similar patterns for equity planning [12,19,21]. To generate groups of participants with similar HLQ strengths and weaknesses (i.e., profiles), hierarchical cluster analysis using Ward’s method for linkage was used. Ward’s method is also known as Ward’s minimum variance method and aims to join elements into clusters while minimizing the variance within clusters [22]. Therefore, the optimal number of clusters was also guided by the aims to minimize the variance within each domain of each cluster (SD <0.6) and ensure the clusters represent different patterns of health literacy strengths and needs. Besides, the demographics and clinical data of subgroups also needed to be considered when determining the optimal number of clusters [12]. The cluster analysis was conducted using SPSS Version 21.

The clusters and related demographic characteristics were then combined to create personal stories/vignettes to represent how a typical person of each cluster accesses and uses health information and services. In addition to the empirical data, in-depth knowledge and experience of working with the local communities from the researchers and public health practitioners were drawn on to develop the vignettes. The vignettes would further be validated at the ideas generation/co-design workshops (see Section 2.2 for details).

### 2.2. Ideas Generation/Co-Design Workshops

The Ophelia ideas generation/co-design workshop is a brainstorm session when the vignettes are presented, and participants are asked to consider ideas/solutions that can be used to help the vignettes. Workshops are usually run separately for community members and for healthcare professionals [12,19,21].

The workshops started with a brief overview of the Ophelia process. It was then clarified that the vignettes to be presented were not real persons but did represent the challenges people in their own community faced in accessing and using health information and services. Participants were encouraged to come up with solutions to help these vignettes based on their personal experiences as a way to harness local wisdom. The following questions were asked to guide the discussion following presentation of each vignette:

Questions for community members:

1. Do you think the person in this story is someone you know from your community?
2. What do you think this person’s problems are?
3. How would you help this person to solve his problems?

Questions for healthcare worker:

1. Do you see clients like this/ do you know people like this?
2. What sort of issues is this person facing?
3. What strategies could you use for an individual like this?
4. What could you do if you had many clients like this in your organization?

These questions are standard questions in the Ophelia process. The first question is used to allow workshop participants to see the characters in the story as someone real and help them identify with the characters. It also serves as a grounded and frontline form of validation of the vignettes. The second question encourages participants to see how health literacy can affect the life of people and the challenges people are facing. Based on these problems, participants can then suggest solutions to help the character on an individual or clinician level. The fourth question is asked to encourage healthcare workers to think of solutions at the organizational or community level so that a holistic approach can be taken to help people with health literacy needs. See Figure 1 for the workshop process to generate co-design solutions.

The first workshop was facilitated by experienced facilitators (RHO, WAA) and attended by the project team. The other workshops were facilitated by the trained project team from the Faculty of Medicine, Ain Shams University. All workshops were audio-recorded with participants’ consent. Two note-takers were also present to record the insights and ideas generated for each vignette. During the discussion, main ideas were written on a flip chart that was used at the end of the workshop to sum up the ideas generated.

#### 2.2.1. Participant Recruitment

Recruitment was conducted by phone to invite community members, including fishermen and wives of fishermen, living in the region, as well as Radaat Refeyat (female nurses of primary health centers serving the fishing communities).

Community members were encouraged to participate through the help of community leaders. In addition, some free medical services to participants or their families preceding the workshops were provided as incentives to participate. They were informed that their suggestions and solutions would help policy makers to improve their living conditions over the coming years. Transportation was provided should participants agree to attend. As a small community and the participants were usually well acquainted with each other, such as neighbours, family and friends, they were generally willing to share their ideas during the workshops. For the one workshop joined by government officials, community members recruited to this workshop were senior fisherman leaders and were familiar with the officials and were comfortable to share their ideas and suggestions. Regarding the Radaat Refeyat, all the teams working in the selected community were invited to participate at the healthcare professionals workshop.

#### 2.2.2. Data Analysis

The solutions generated from the workshops were first coded across three theoretical themes by DGS:

1. Governmental level
2. Non-government organizations (NGOs) and fishermen syndicates level
3. Individual level

Further thematic analysis based on the content was undertaken by the research team and reviewed by the principal investigator (WAA), and the international collaborator (RHO). Thematic analysis is a reflective process. To ensure that the conclusions drawn accurately reflected the content of the workshops, two authors (SAH and DGS) separately coded all the workshop data using an iterative constant comparative method [23]. The process involved three stages which included initial coding, focused coding to reduce overlap and redundancy of coding, and theoretical coding using the themes identified above [24]. Two cross-checks to compare emerging themes was performed to assess for the accuracy of inferences at each stage. Where a difference was found, the authors were asked to demonstrate from the raw data how their interpretation was determined until agreement was reached. A pragmatic approach was adopted when drawing final inferences from this study, with a focus on the development of useful knowledge directly related to health literacy for the fishermen communities. Final inferences were verified by NSM and WAA. To ensure consistency with the co-design process, the data were then referred back to those who attended the workshops to provide respondent validation.

## 3. Results

The cluster analysis was undertaken for a subset of the health literacy survey, with 178 survey participants. Seven clusters were identified with different health literacy profiles. Cluster A had the highest scores across all nine scales compared to the other clusters. The other clusters all had some higher scores in certain scales but lower scores in other scales, indicating varying strengths and weaknesses in different health literacy domains. See Table 2. The average age of Cluster A was 40.6 and 65% of the people in this cluster were male. About 55% of them were fishermen, with 95% lived in a family and 18% were illiterate. The illiterates represented 20%, 63%, 59%, 57%, 62%, and 80% of Clusters B–G respectively (see Table 3 for other demographic details). Seven vignettes were developed for the seven clusters and were translated into simple Arabic for presentation at the workshops. Refer to Table 4 for an example of a vignette from Cluster G.

Four workshops were held during 2019 and in early 2020. Almost all invited participants attended the sessions. People who did not participate had personal reasons rather than being unwilling to participate. A total of 25 participants joined the first two workshops. Participants included 17 fishermen, five representatives of fishermen syndicate, two representatives of the Health Directorate, Ministry of Health from the same governorate and one representative of the Cooperative Union of Egyptian Water Resources. The mean age of the 17 fishermen was 50 and all of them did not complete secondary school. Each of them had at least 25 years of experience in the fishing industry. Two of the fishermen had cardiovascular disease. Workshop 3 included 20 Radaat Refeyat (mean age: 40) and workshop 4 was attended by 22 wives of fishermen (mean age: 45). Ten of the 22 wives of fishermen reported having two or more chronic conditions.

With the first question about whether participants recognized this kind of person in their community, participants universally and strongly agreed that the vignettes were common in their community. This provided grounded validation that the vignettes presented were appropriately developed to represent the experience of local community members when accessing and using health information and services.

Across the workshops, a total of 80 intervention ideas to improve the care and services for the vignettes were generated. The number of ideas ranged from 10 to 25 per workshop. Five themes with 15 general strategies emerged from the analysis and were then grouped across the three theoretical themes of governmental level, NGO and syndicates level, and individual level (See Table 5).

Theme 1: Enhancing education among fishing communities.

This theme included ideas at both governmental level and NGOs and fishermen syndicates level. Participants from both community members and healthcare workers workshops agreed that the four main requirements for enhancing education among fishing communities were:

(1) Establishing national programs for literacy and adult education that suit their working career.
  “providing a suitable educational program for adults that suit them will even enable them to acquire a driving license and enable him to stand on solid floor”—Workshop 2
(2) Initiating a basic educational course through fishermen syndicate at a reasonable and affordable price.
  “Fishermen syndicate knows our basic needs and the level of education that we should start from”—Workshop 1
(3) Health education sessions in the primary health centers and through home visits that can be conducted by Raedat Refyeat.
  “We usually conduct home visits and know all the fishermen housewives through the vaccination campaigns which could be good opportunity to deliver health education tips for them”—Workshop 3
(4) Establishing awareness campaign using the mass media (television) to provide health education regarding different health topics such as family planning, healthy diet and how to quit risky behaviors such as smoking.
  “TV is on the whole day, if it can be an educational source instead of entertainment for some time, it will help us a lot”—Workshop 4

Theme 2: Provide good quality health care services.

Participants reported facing difficulties in accessing health services at the right time or simply unable to reach acceptable quality care. The following strategies, including at the governmental level and NGOs and fishermen syndicates levels, were suggested:

(5) Increase number of well-trained physicians and specialists in government hospitals.
  “Usually fresh graduated doctors are found, and we face difficulty reaching senior expert in the field”—Workshop 1
(6) Healthcare providers to undergo effective communication skills training.
  “Sometimes we don’t feel that the doctor understands our suffering and in many times I don’t understand the doctor’s instructions and hence don’t take or follow his prescriptions”—Workshop 4
(7) Enhance service quality to ensure customer satisfaction through continuous monitoring and auditing of service providers.
  “Having a frequent audit visit will ensure better service to achieve our satisfaction”—Workshop 1
(8) Frequent medical convoys from non-governmental organizations that provide both diagnostic and treatment services.
  “NGOs can reach us easily and communicate with us effectively and even provide all the required medications for us immediately after diagnosis”—Workshop 2 and 4

Theme 3: Financial support for health.

Participants in the workshop identified that financial support as the main enabler to access health service and ensure better health related quality of life. Three overall strategies involving both government, NGOs and fishermen syndicates were suggested:

(9) Pension for fishermen from governmental and non-governmental sources.
  “Although we face many accidents during our fishing career, yet we don’t have any pension later. We don’t have a fixed income, hence ensuring food on the table is number1 priority”—Workshops 1 and 2
(10) Financial support for starting small projects that can be managed at home.
  “We can work from home through small projects as making clothes, but we need a sum of money to start. If we can have this, we will help our husbands”—Workshop 4
  “Most of fishermen wives are talented and have many skills but they need support to start their dreams”—Workshop 3
(11) Health insurance cover for the fishermen and their families.
  “There is no health insurance at all, so we seek doctors in late stages and never at an early stage”—Workshop 1 and 2

Theme 4: Social support for health.

Wives of fishermen mentioned that social support will encourage them to acquire healthy behaviors, which could be undertaken by NGOs and fishermen syndicates. The following strategies were suggested:

(12) Availability of clubs for sports and exercise to improve health and enable weight reduction.
  “We don’t have a place to walk or even for our children to practice any sport, we are sitting all the time in our homes and get fat”—Workshop 4
(13) Group physical therapy and education sessions for people going through the same health experience to allow for sharing of information, resources, and strategies from others’ lived experience.
  “When we talk together and hear others similar situation and their success, we feel that there is a hope and we can change our habits”—Workshop 4

Theme 5: Promoting better health-related quality of life among fishermen.

This theme was mainly targeted at the individuals. Radaat Refeyat suggested that individuals could promote better quality of life through the following two strategies:

(14) Female household members can serve nutritious diet and keep their family healthier.
  “If every mother prevented junk food and served salad daily—especially that television now provides many healthy food channels that can support a lot—all members of her family will acquire healthy diet behaviors”—Workshop 4
(15) Walking twice daily for half an hour as regular exercise.
  “We live in villages, everything is near, and the air is clean that encourages walking”—Workshop 4

## 4. Discussion

This study undertook the Ophelia process, a co-design approach, to identify potential interventions to improve health and equity outcomes for the fishing communities in the Borollos Lake region of Northern Egypt. The results of 15 key strategies across five themes indicate that the Ophelia process can be effectively applied in a low resource setting [25]. By following the Ophelia process, it is expected that this research can be reproduced in other similar community settings.

The health literacy survey found that most people scored at the ‘disagree’ or ‘difficult’/’very difficult’ end of the scales even for people with higher income or education, particularly for scales about having enough information, being able to find good information, being able to appraise information and actively managing their health while they tend to score higher in social support and communicating with health professionals, reflecting a culture of communal practice [5]. Hence, health literacy development in community settings such as the unique rural fishing communities of this study requires an understanding of the ways in which families, friends, and peers interact and how these social networks influence how people think and act in relation to their health [26].

Although many of the participants had limited education, this study engaged them in the design and development of locally relevant programs for their own community. Instead of providing participants with statistical data, they heard stories of people whom they recognized. This was useful because they could relate to the same problems, challenges, and limited resources, as well as understand the negative impacts on their health. In spite of limited education, the results showed that participants did have deep understanding of their own contexts and can generate realistic low-cost local solutions. Through meaningful community participation irrespective of people’s education level or social status, this study has captured the essence of communal culture common in rural communities.

This study used the HLQ to understand the health literacy strengths and weaknesses of people living in low resources setting. Instead of focusing only on health-related literacy or numerical skills as in other health literacy studies, or simplistic health literacy data expressed as inadequate vs adequate, this study revealed nuanced challenges people have when accessing and using health information and services. This study also found that people could have diverse health literacy needs and a one-size-fits-all approach is inappropriate to address these needs.

By using the Ophelia process to engage with and provide a voice for the communities through the ideas generation/co-design workshops, the unique limitations and resources available in the communities can be identified to support the health literacy needs of people living in these communities. For example, the two fishermen workshops indicated that organized literacy programs must suit their working hours. Both fishermen and Radaat Refeyat had identified television as the appropriate channel for delivering health education, unlike the many digital interventions proposed in high-income countries these days. Walking should be promoted as a regular exercise, suggested by the wives of fishermen, because they pointed to the clean and fresh air in the villages. The wives of fishermen also noted that children did not have access to any facilities to exercise and therefore sporting facilities were required. By harnessing local knowledge and wisdom, uptake of interventions generated from the co-design process will likely be higher as they suit the special needs or environment of the communities.

Two of the strategies commonly raised at different workshops were ‘Increasing the numbers of medical convoys from NGOs and co-ordination between different NGOs to provide regular services’ (proposed at the fishermen and Radaat Refeyat workshops) and ‘Financial support for starting small projects that can be managed at home’ (proposed at the wives of fishermen and Radaat Refeyat workshops). On the other hand, the other strategies were uniquely suggested by different groups, representing the different perspectives of fishermen, wives of fishermen and Radaat Refeyat in meeting the health literacy needs of the fishing communities. The findings indicate that both community members and healthcare professionals are important players in the co-design process.

The overall HLQ mean score indicated weaknesses around health information (Scales 2, 5, 8, and 9) while the demographics further showed that 37.1% of the sample were illiterate [5]. These weaknesses were also identified among the majority of the sample (71.9%) from Clusters C–G. In response to the vignettes representing these data, the strategies such as health education sessions and awareness campaigns were suggested. These ideas echoed the recommendation of an earlier research brief to organize training programs to improve the skills, knowledge and behaviour among the Kafr El Sheikh population [27].

The difficulties in navigating the healthcare system (Scale 7), strongly expressed in Clusters C–G, led to the idea of frequent medical convoys from NGOs to provide regular healthcare services. The need for convoys is in fact urgent because convoys can complement the diagnostic and medication services of hospitals, primary healthcare centers and clinics. As such, this strategy is an opportunity to provide equal access to health services in the region.

While strengths in social support are found in the overall sample, reflecting the communal culture of the fishing communities, the cluster analysis identified two clusters (Clusters E and G) that did not seem to have adequate social support. These two clusters represented a quarter (25.8%) of the sample. By using the Ophelia process to ensure health equity and that disadvantaged groups were not overlooked, the needs of the people in these two clusters were reflected in the vignettes. The strategy of ‘Group sessions to allow for sharing of information, resources, and strategies from others’ lived experience’ will likely meet the need for social support among people of Clusters E and G.

The financial needs voiced by the fishermen and wives of fishermen represented real-life issues that need to be tackled in order to achieve better health outcomes. Fishing is an unpredictable occupation and the tough environment leads fishermen to make their work a priority to secure income instead of making their health a priority [1,5]. Therefore, poverty is a problem that needs to be addressed. Without financial security, such as a pension, health insurance cover, or support for small projects proposed at the workshops, fishermen will continue to prioritize work instead of their health.

The range of strategies identified in the Ophelia workshops showed that meeting health literacy needs should not only be the responsibility of the individuals. The results have identified action areas for government, NGOs, fishermen syndicates, and families and communities as collaborators in the effort of health literacy development. As many individuals at this fishing community are either illiterate or have limited education, individuals may need to receive education and take action with family to enhance health. However, ideas involving governments and NGOs were also generated based on local wisdom in this study. While education is closely related to improving health literacy of communities and several reports originated from developing countries highlighted the positive impact of education on health and pointed out that a lack of literacy contributes significantly to disease burden, others suggested that a higher level of education or literacy does not necessarily ensure a high level of health literacy [28]. As such, ideas from this study, such as involving governments and NGOs to improve health services, deliver quality care, establish enabling environments, and provide financial support, are needed to achieve better health and equity outcomes for the community.

Egypt has gone through several steps to promote health equity, which includes providing care that does not vary in quality because of personal characteristics such as gender, ethnicity, geographic location, and socioeconomic status. This is being achieved through constructing new hospitals that are well equipped, allocating more budget resources to health, supporting primary health care, conducting convoys that provide medical examination and treatment, and maintaining several presidential initiatives, such as raising awareness against hepatitis C, early diagnosis of breast cancer, and non-communicable diseases.

This study added another step towards improvement of health equity in Egypt. The intervention ideas generated through community participation lay the foundation for the next phase of the Ophelia process to select and implement interventions. Based on the findings from this study, several intervention ideas were initiated, mainly focused on the establishment of health education in different health fields with special consideration to diet and nutrition as well as healthy lifestyles and self-care. A report was also submitted to government authorities that included the community’s ideas and suggestions.

### Limitations

A limitation of this study was that some groups of community leaders in governmental organizations and NGOs did not participate in the ideas generation/co-design workshops. Recruitment of community members workshop participants also did not undergo formal sampling processes. This may have affected the representativeness of community members. Another limitation was that only researchers and healthcare workers were involved in the development of vignettes but not community members. However, the universal agreement that the vignettes were well-recognized during the workshops indicated the vignettes did capture the lived experience of people in their community. A further limitation to the co-design strategy was that workshop participants or community members had yet to be involved in the subsequent process of intervention ideas implementation. Future co-design studies should consider engaging community members in the design, development, and even refinement of health literacy interventions.

## 5. Conclusions

Fishing communities in low resource settings are at risk of lower health literacy and hence poor health. This study applied the Ophelia process to understand health literacy needs and used local wisdom to generate intervention ideas to address these needs. A total of 15 strategies across five themes that suit local needs were developed. Health literacy actions were identified for government, NGOs, fishing syndicates, fishermen and their families, providing a holistic approach to promote health literacy development for the community. By harnessing local knowledge and wisdom, practical and doable solutions that matched the special needs and environment of the community were generated. These ideas are ready to be selected, tested, and implemented to improve health and equity outcomes for the fishing community.

## Figures and Tables

**Table 1:** Health Literacy Questionnaire (HLQ) scores of people from fishing communities in Borollos Lake region, Northern Egypt.

HLQ Scale	Mean (SD) [95% CI]
	Range: 1 (lowest)–4 (highest) *
1. Feeling understood and supported by healthcare providers	2.51 (0.83) [2.43–2.58]
2. Having sufficient information to manage my health	2.23 (0.76) [2.16–2.30]
3. Actively managing my health	2.37 (0.75) [2.30–2.44]
4. Social support for health	2.95 (0.69) [2.89–3.03]
5. Appraisal of health information	2.37 (0.79) [2.29–2.44]
	Range: 1 (lowest)–5 (highest) ^
6. Ability to actively engage with healthcare providers	3.50 (0.96) [3.41–3.59]
7. Navigating the healthcare system	3.11 (1.03) [3.01–3.21]
8. Ability to find good health information	2.78 (1.10) [2.67–2.88]
9. Understand health information well enough to know what to do	3.26 (0.88) [3.18–3.35]

SD = standard deviation; CI = confidence interval. * 1 = strongly disagree, 2 = disagree, 3 = agree, 4 = strongly agree. ^ 1 = cannot do or usually difficult, 2 = very difficult, 3 = quite difficult, 4 = easy, 5 = very easy.

**Table 2:** Health literacy seven cluster solution.

Cluster			A	B	C	D	E	F	G
Cluster N			20	30	52	22	21	8	25
% of sample in cluster			11.2	16.9	29.2	12.4	11.8	4.5	14.0
Mean age			40.6	41.5	43.8	45.2	39.0	39.1	49.3
1. Feeling understood and supported by healthcare providers	Mean Score	Range: 1 (lowest)–4 (highest)	3.75	2.02	2.85	2.85	2.15	1.31	1.18
2. Having sufficient information to manage my health			3.42	2.66	2.54	1.74	2.15	1.06	1.07
3. Actively managing my health			3.27	3.10	2.91	2.31	2.28	3.55	1.90
4. Social support for health			3.73	3.46	3.16	3.15	2.20	3.75	2.87
5. Appraisal of health information			3.63	3.12	2.75	1.86	2.26	1.10	1.08
6. Ability to actively engage with healthcare providers		Range: 1 (lowest)–5 (highest)	4.68	4.35	4.04	3.51	2.70	2.90	2.66
7. Navigating the healthcare system			4.32	3.97	3.38	2.89	2.48	1.92	1.54
8. Ability to find good health information			4.34	4.04	2.97	1.93	2.39	1.57	1.16
9. Understand health information well enough to know what to do			4.36	4.35	3.23	2.79	2.53	3.20	2.60
1. Feeling understood and supported by healthcare providers	Standard Deviation		0.30	0.85	0.66	0.64	0.76	0.35	0.41
2. Having sufficient information to manage my health			0.42	0.79	0.49	0.50	0.59	0.18	0.18
3. Actively managing my health			0.48	0.86	0.40	0.47	0.64	0.42	0.69
4. Social support for health			0.29	0.44	0.48	0.38	0.54	0.41	0.47
5. Appraisal of health information			0.33	0.71	0.52	0.50	0.56	0.19	0.24
6. Ability to actively engage with healthcare providers			0.26	0.53	0.50	0.61	0.73	0.81	0.66
7. Navigating the healthcare system			0.47	0.67	0.53	0.56	0.66	0.68	0.66
8. Ability to find good health information			0.43	0.69	0.65	0.51	0.53	0.31	0.29
9. Understand health information well enough to know what to do			0.49	0.59	0.62	0.53	0.69	0.83	0.61

Note: The scores are highlighted using the traffic light system of colour coding as recommended in the Ophelia process. Cells coloured in green represented higher scores, the range of yellow represents medium scores and red indicates lower scores.

**Table 3:** Demographic characteristics of clusters.

Cluster Group	Mean Age	% Male	% Fisherman	% Housewife	% Other	% Lives in a Family	% Illiterate	% Low Income	% Average Income	% High Income
A	40.6	65	55	20	25	95	18	26	58	16
B	41.53	53	27	40	33	100	20	40	37	23
C	43.79	46	42	38	19	96	63	82	16	2
D	45.18	55	50	18	32	100	59	82	9	9
E	39	62	52	33	14	86	57	76	19	5
F	39.12	62	62	38	0	100	62	0	25	75
G	49.32	92	88	8	4	96	80	24	60	16

**Table 4:** Example of a vignette (Cluster G).

**% of Sample in Cluster G**		**14**	Hassan is a Male fisherman aged 49. Illiterate, did 1–2 years of schooling. Moderate health, has some inflammatory disease. He is a smoker and quite overweight. Not an internet user, moderate income. Very low health literacy, struggles with most aspects. Has some social support for health, and his other relative strengths are in being able to talk with health providers and understand health information, but these are still low. Would have great difficulty finding information—he doesn’t feel he has enough information but as his health is not at all a priority to him, he may not see this as a problem.
1. Feeling understood and supported by healthcare providers	Score range: 1 (lowest)–4 (highest)	1.18	
2. Have sufficient information to manage my health		1.07	
3. Actively managing my health		1.9	
4. Social support for health		2.87	
5. Appraisal of health information		1.08	
6. Actively engage with healthcare providers	Score range: 1 (lowest)–5 (highest)	2.66	
7. Ability to navigate the healthcare system		1.54	
8. Ability to find good health information		1.16	
9. Understand health information well enough to know what to do		2.6	

Note: The scores are highlighted using the traffic light system of colour coding as recommended in the Ophelia process. Cells coloured in green represented higher scores, the range of yellow represents medium scores and red indicates lower scores.

**Table 5:** Workshop thematic analysis.

Theoretical Theme	Emergent Theme	Issue	Solutions	Issue/Solutions Raised In		
				Fishermen Workshop	Wives of Fishermen Workshop	Raedat Refyeat Workshop
**Governmental level**	***Theme 1: Enhancing education among fishing communities***	Literacy classes are usually in the morning and at fixed time	Establish literacy classes on weekends of the fishermen and afternoon classes	✓		
			Educational sessions in primary health centers visited by wives of fishermen		✓	
			Provide health educational tips during home visits done by Raedat Refyeat		✓	
			Health Educational programs on television that target pressing issues (e.g., family planning, personal hygiene)			✓
	***Theme 2:*** ***Provide good quality health care services***	Lack of specialized staff in certain specialties	Increase number of trained specialized health care workers	✓		
		Fishermen are not satisfied with service provided	Training programs for doctors and physicians to ensure effective communication			✓
			Monitoring staff performance and conducting frequent audit visits of health care services	✓		
	***Theme 3:*** ***Financial support for health***	Fishermen (private carrier) do not have salaries and no pension	Fixed pension should be provided or social security after fishermen retire	✓		
		Fishermen are unable to pay for the health services	Health insurance cover should encompass the fishing communities.	✓		
**NGOs and Fishermen syndicates**	***Theme 1:*** ***Enhancing education among fishing communities***	Lack of basic education	Assigning an educational course at their yearly vacation and provide certificate of pass	✓		
	***Theme 2:*** ***Provide good quality health care services***	Most fishing communities suffer from chronic diseases that needs frequent follow up	Increase numbers of medical convoys from NGOs and co-ordination between different NGOs to provide regular services	✓		✓
			Provision of screening services and provision of medication supply especially for chronic cases		✓	
	***Theme 3:*** ***Financial support for health***	All fishermen wives do not work although they are capable	Financial support for starting small projects that can be managed at home		✓	✓
	***Theme 4:*** ***Social support for health***	No clubs or spaces to practice any kind of sport or walking	NGOs should collaborate with locals to build youth centers and clubs for the elderly			✓
			Health promotion sessions on how to keep fit and should include diet as well as simple physical exercises.			✓
**Individual level**	***Theme 5:*** ***Promoting better health-related Quality of life among fishermen***	Lack of exercise	Fishermen should include walking for half an hour twice weekly to stay healthy			✓
		Fishermen do not follow healthy dietary pattern	Wives of fishermen should be encouraged to avoid junk food and cook healthy food daily, such as green vegetables and fresh salad			✓

Levels are in bold. Themes are in bold and italics.

## Data Availability

The data presented in this study are available in Supplementary Material.

## Supplementary Materials

The following are available online at https://www.mdpi.com/article/10.3390/ijerph18094518/s1, Table S1: Data.
